# Inflammasome Activation in Pulmonary Arterial Hypertension

**DOI:** 10.3389/fmed.2021.826557

**Published:** 2022-01-13

**Authors:** Anna Foley, Benjamin E. Steinberg, Neil M. Goldenberg

**Affiliations:** ^1^Department of Physiology, University of Toronto, Toronto, ON, Canada; ^2^Department of Anesthesia and Pain Medicine, The Hospital for Sick Children, The University of Toronto, Toronto, ON, Canada

**Keywords:** pulmonary hypertension, inflammasome, macrophage, endothelial, vascular remodeling

## Abstract

Inflammasomes are multi-protein complexes that sense both infectious and sterile inflammatory stimuli, launching a cascade of responses to propagate danger signaling throughout an affected tissue. Recent studies have implicated inflammasome activation in a variety of pulmonary diseases, including pulmonary arterial hypertension (PAH). Indeed, the end-products of inflammasome activation, including interleukin (IL)-1β, IL-18, and lytic cell death (“pyroptosis”) are all key biomarkers of PAH, and are potentially therapeutic targets for human disease. This review summarizes current knowledge of inflammasome activation in immune and vascular cells of the lung, with a focus on the role of these pathways in the pathogenesis of PAH. Special emphasis is placed on areas of potential drug development focused on inhibition of inflammasomes and their downstream effectors.

## The Inflammasome

**Figure 1:**
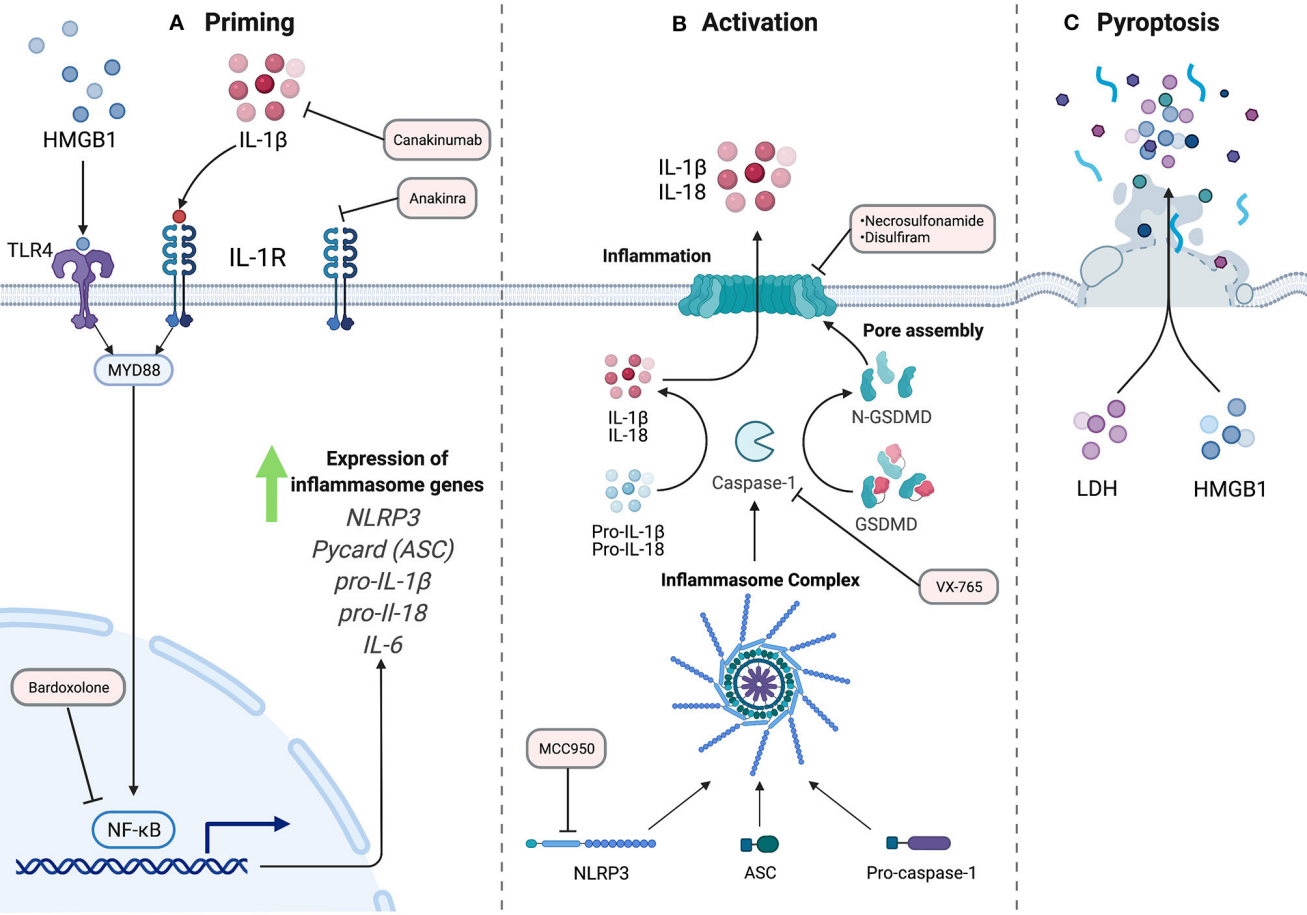
The NLRP3 Inflammasome Pathway. A two-signal model for NLRP3 inflammasome activation. **(A)** Priming, signal 1, is accomplished by extracellular ligands through receptors such as Toll-like receptors. This results in activation of transcription factor NF-κB and subsequent upregulation of inflammasome machinery and pro-inflammatory components. **(B)** Activation, signal 2, which can be achieved by a variety of stimuli resulting in the assembly of the inflammasome complex. Activated caspase-1 cleaves inflammatory cytokines, including pro-IL-1B and pro-IL-18 into their active forms. Additionally, GSDMD is cleaved to allow for N-terminal oligomerization and pore formation at the plasma membrane for release of mature cytokines. **(C)** In some conditions, the cell can undergo pro-inflammatory cell death termed pyroptosis, with frank rupture of the cell, and subsequent release of large macromolecules into the extracellular space, including danger-associated molecular patterns like HMGB1, and pro-inflammatory molecules like ATP, histones, and nucleic acids. The red rectangles indicate steps in the inflammasome pathway that can be targeted pharmacologically, and current agents that work at these steps.

Inflammasomes are multi-protein complexes involved in sensing both endogenous and exogenous cellular stress (Figure 1) (1). Inflammasomes can respond to a variety of pathogen-associated molecular patters (PAMP's) and damage-associated molecular patters (DAMP's) based on the identity of the pattern recognition receptor (PRR) within the complex (2). In general, inflammasome pathways consist of a receptor, adaptor, and effectors. Five receptor proteins have been confirmed to form the “canonical” inflammasomes to date: nucleotide-binding oligomerization domain (NOD), leucine-rich repeat (LRR)-containing proteins (NLR) family members NLRP1, NLRP3, and NLRC4 as well as the proteins absent in melanoma 2 (AIM2) and pyrin (3). In addition to the canonical pathways, there is also the non-canonical pathway in which caspase-4/5 in humans and caspase-11 in mice are activated directly by intracellular triggers (4). Recognition of inflammatory stimuli results in the activation and oligomerization of the complex that often includes the adaptor protein, apoptosis associated speck-like protein (ASC). The oligomerization with ASC, which contains a caspase recruitment domain (CARD), establishes the activation platform for the pro-inflammatory caspase that facilitates the formation of the functional inflammasome complex (5). The NLRP3 inflammasome is the best characterized and will be the pathway of focus in this review.

The formation of the NLRP3 inflammasome complex allows the proximity-induced autocatalytic activation of pro-caspase-1 to cleaved caspase-1. The role of caspase-1 downstream is the cleavage of cytokines such as pro-IL-1β and pro-IL-18 to their biologically active forms. Caspase-1 additionally cleaves gasdermin D (GSDMD), a protein in which the N terminal subunits assemble into a multi-unit complex to form a plasma membrane pore. The inner diameter of this pore is 21.5 nm and is the main route of egress for mature cytosolic cytokines into the extracellular space (6). Largely non-selective, the GSDMD pore allows free passage of appropriately sized molecules between the cytosol and the extracellular space. In some conditions, these pores lead to lytic cell death, termed pyroptosis (7). This pro-inflammatory cell death pathway requires the protein ninjurin-1 (NINJ1) through a mechanism that has yet to be defined in detail (8). Following cell membrane rupture, larger proteins such as the potent signaling molecule high-mobility group box 1 (HMGB1) are released into the extracellular environment, propagating inflammatory signals to neighboring cells (9). While this process is best described in immune cells, evidence exists for inflammasome activation in non-immune cells as well, including vascular endothelial cells (10–12).

### Triggers and Effectors

The most commonly described stimulus for inflammasome activation *in vitro* involves two steps: a priming step followed by an activation trigger (13). The priming signal is accomplished by extracellular ligands for Toll-like receptors (TLRs), such as bacterial lipopolysaccharide, and results in the activation of the transcription factor NF-κB. This signal upregulates NLRP3, pro- IL-1β, and other inflammasome components, as those proteins are not expressed at a sufficient level in a resting cell for activation (13).

The NLRP3 inflammasome is activated by a wide variety of stimuli that are different in both their physical structure and their chemical nature. As a result of these differences, it is suspected that these diverse stimuli all converge in a common cellular event that activates the inflammasome (5). Potassium efflux through the plasma membrane is critical to multiple NLRP3 inflammasome activators such as bacterial pore-forming toxins and extracellular ATP. Effectively serving as a type of DAMP, extracellular ATP activates the purinergic P2X receptor 7 (P2X7), which is an ion channel selective for Na^+^, K^+^, and Ca^2+^ ions. The ATP-gated receptor initiates the depletion of the K^+^ concentration in the cell (14). Furthermore, the decrease of K^+^ concentration in the cell alone is sufficient for inflammasome activation (15). The potassium sensor for the NLRP3 pathway is still an area of investigation. However, there is some work that demonstrates NEK7, of the NIMA-related kinase family, as an essential protein that acts downstream of potassium efflux leading to the association of NEK7 with NLRP3 for the assembly of the inflammasome and subsequent activation (16, 17).

Mitochondrial dysfunction, resulting in production of mitochondrial reactive oxygen species (mtROS) and release of mitochondrial DNA, is considered another activator of the NLRP3 pathway. Gross et al. demonstrated that mtROS led to NLRP3 activation in a K^+^ independent mechanism (18). Mitochondrial DNA, which can be oxidized by mtROS showed similar findings of inflammasome activation in mouse bone marrow derived macrophages (BMDM's) (19). The link between mitochondria and inflammasomes is of special interest given the known role of mitochondrial dysfunction in PAH. Indeed, mitochondrial DNA can directly activate both the NLRP3 and AIM2 inflammasomes (20). The proliferative phenotype of PAH can be at least partially attributed to mitochondrial metabolism. The Warburg phenomenon—glycolysis and lactate metabolism even in the presence of oxygen—allows cells to maintain a highly proliferative state [reviewed in (21)]. Furthermore, mitochondrial fission stimulates fibroblast proliferation in the vasculature and right ventricle, driving adverse remodeling (22). Interestingly, these phenomena link to both activation and regulation of inflammasomes. The oligomerization of GSDMD is controlled by the Ragulator-Rag complex upstream of the master metabolic regulator, mTOR (23). Linking through the mitochondria, GSDMD pore formation was enhanced by mitochondrial dysfunction, as seen through loss of mitochondrial membrane potential and mROS production (23). Together, these data suggest that conditions favoring mitochondrial dysfunction, as seen in PAH, favor inflammasome activation. Additionally, treatments targeting metabolic dysfunction, such as metformin, are under intensive investigation clinically (24).

## Pulmonary Arterial Hypertension

**Figure 2:**
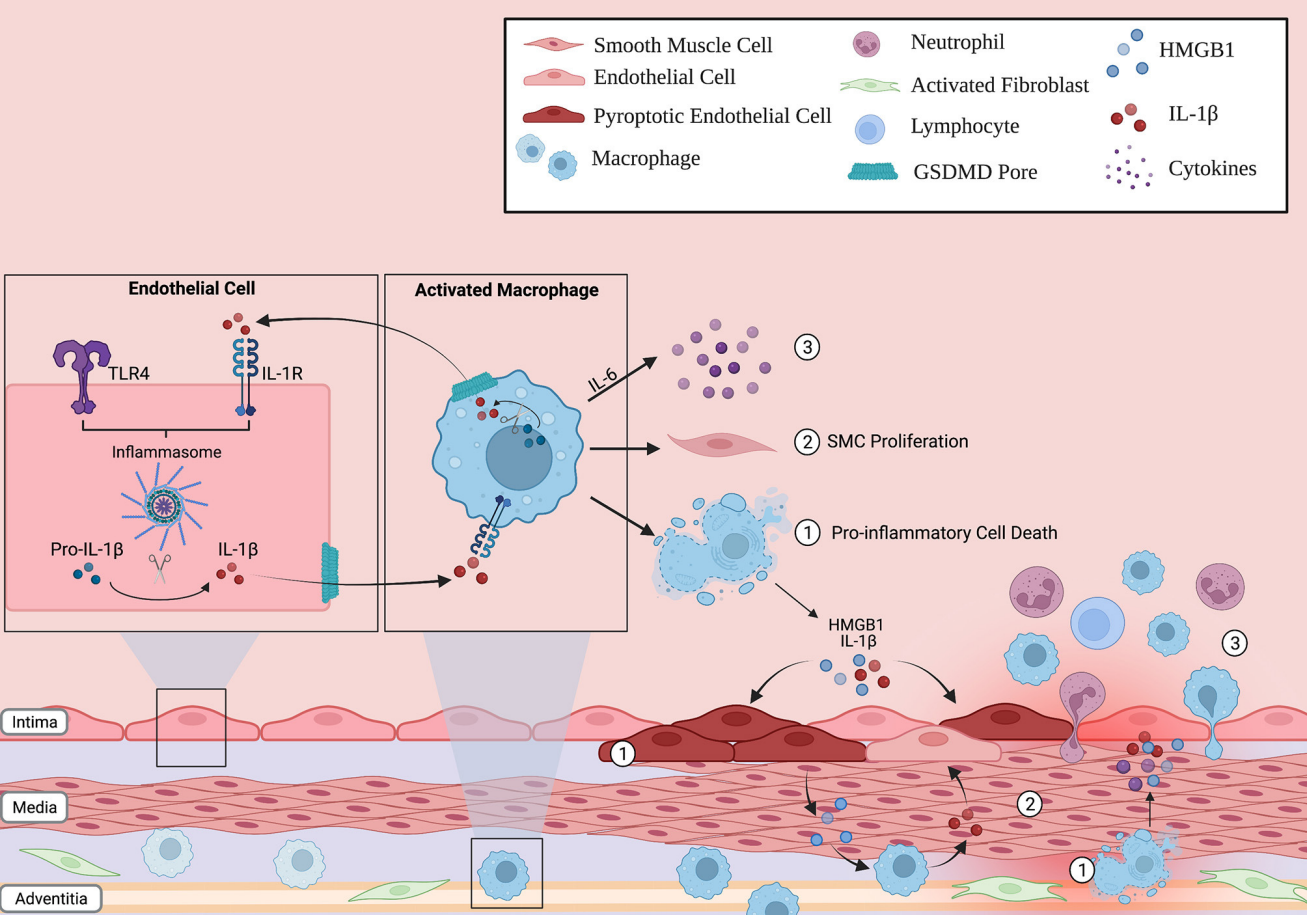
Inflammasome action in the lung vascular environment. Immune cells (such as macrophages) and non-immune cells (endothelial cells) can respond to stress, activate the inflammasome pathway and undergo pyroptosis. The release of pro-inflammatory cytokines through the GSDMD pore, such as IL-1B, can propagate inflammation in neighboring cells by inducing their own transcription in a paracrine fashion. As such, the innate immune response can be amplified through the lung vasculature. In addition, these activated cells can (1) release other potent signaling molecules, such as HMGB1, into the environment following lytic cell death, (2) increase smooth muscle cell (SMC) proliferation in the medial layer of the vessel, and (3) release cytokines such as IL-6 that recruit other inflammatory cells to the vessel wall. Overall, this creates a vicious inflammatory cycle that, over time, contributes to the characteristic pulmonary vascular remodeling seen in PAH patients.

Pulmonary Hypertension (PH) is highly fatal disease defined by a mean pulmonary arterial pressure >20 mmHg at rest (25). Pulmonary Arterial Hypertension (PAH) is classified by the World Health organization as Group 1 PH, and is characterized by elevated pulmonary vascular resistance and lung vascular remodeling (26). This remodeling consists of alterations in the structure and growth of all three layers of the vessel—the intima, media and adventitia—resulting in the high pulmonary vascular resistance, right ventricular failure and ultimately death (27). In recent years, the importance of inflammation in the pathogenesis of pulmonary hypertension has received greater attention. The immune cell infiltrates observed in diseased vessels in both human and animal models of PH include cells such as T cells, B cells, neutrophils, macrophages, and others (27). In addition to the presence of autoantibodies, both PH animal models and PAH patients have shown high levels of cytokines that are relevant to the effectors of inflammasome activation (28). The presence of inflammasome products in PAH patients, as well as the efficacy of cytokine blockade in treating PAH in patients and animal models have provided the rationale for targeting inflammasomes in PAH therapy. In the remaining sections, we will review pre-clinical and clinical evidence for blocking inflammasomes in PAH, and will suggest future targets for such a strategy (Figure 2).

### Pre-clinical Evidence

The NLRP3 inflammasome and its downstream components have been investigated in the context of many pulmonary diseases such as COPD, asthma, cystic fibrosis and pulmonary fibrosis (29–32). There are multiple lines of *in vitro* and animal model evidence implicating inflammasomes in the pathogenesis of PAH (Table 1). Following chronic hypoxia, mice lacking the inflammasome adaptor, ASC, were protected from elevated right ventricular systolic pressure (RVSP) and right ventricular hypertrophy (33). Furthermore, ASC^−/−^ mice demonstrated no pulmonary increase in caspase-1, IL-18, or IL-1β abundance, in contrast to wildtype controls. Surprisingly, NLRP3 knockout mice developed disease to a similar extent as wildtype, with no significant difference in right ventricular systolic pressure (RVSP) or cytokine levels (33). These data are consistent with possible compensation for loss of an individual inflammasome type by others, since loss of NLRP3 has no effect, but loss of ASC which is required for most inflammasomes, is protective.

**Table 1:** Pre-clinical and clinical evidence for inflammasomes in PAH.

**Molecule**	**Pre-clinical**	**Clinical**	**Drug target**	**References**
NLRP3	*NLRP3^−/−^* mice were not protected from hypoxic PH	NS	MCC950	(33, 53–55)
ASC	*ASC^−/−^* mice protected from hypoxic PH and RV hypertrophy, with decreased IL-1β and IL-6	NS	None	(33)
Caspase-1	• Increased lung expression of active caspase-1 in chronic hypoxic mice • Caspase-1 induces smooth muscle cell proliferation	NS	VX-765	(33, 42, 56)
Gasdermin D	NS	NS	Necrosulfonamide Disulfiram	(57, 58)
IL-1β/IL-1R	• IL-1β stimulates smooth muscle cell proliferation • *IL-1R^−/−^* mice protected from chronic hypoxic PH	Increased serum IL-1 correlates with worse outcomes in PAH	Anakinra Canakinumab	(34, 49–51)
NF-κB	Induces expression of IL-6 and drives PH in mice	NF-κB inhibitor in Phase 3 clinical trial	Bardoxolone	(37, 52)
HMGB1	• Anti-HMGB1 antibody rescues PH in Sugen-hypoxia and monocrotaline rats • Stimulates pulmonary endothelial cell proliferation	Increased HMGB1 in idiopathic and CHD-associated PAH	None	(35, 36, 48)

*This table summarizes current pre-clinical and clinical evidence for the involvement of inflammasomes in PAH. NS, not studied. See text for details*.

Downstream of inflammasome activation, IL-1β and its receptor, IL-1R, are involved in the pathogenesis of PH in animal models. Within hours of exposure to hypoxia, IL-1β, IL-1R, and the IL-1R adaptor, MyD88, are upregulated in the lungs of mice (34). Knockout mice lacking either MyD88 or IL-1R are protected from hypoxic PH, as are those treated with the IL-1R receptor blocker, anakinra (34). Either genetic or pharmacological blockade of IL-1R reduced macrophage infiltration into hypoxic mouse lungs. In cultured pulmonary artery smooth muscle cells, IL-1β stimulated proliferation in an IL-1R and MyD88 dependent fashion (34). Together, these results indicate a key role for signaling through IL-1R in PH. Interestingly, blockade of IL-1β also has the beneficial effect of inhibiting upstream inflammasomes. There are several possibilities for how this effect evolves. There is potential for upstream negative feedback, but more intriguingly, the role of MyD88 and NF-κB sets up the possibility of breaking a cycle of self-amplification of inflammasome signaling. While IL-1β is a product of inflammasome activation, its receptor engages MyD88 and NF-κB, in a similar manner to TLR. Therefore, IL-1β can then induce its own upregulation in both an autocrine and paracrine manner. Blockade of the IL-1R, hence, can break this cycle, leading to profound dampening of this system. Indeed, the involvement of TLR signaling in inflammasome pathways opens a variety of potential routes to targeting important mechanisms of PAH development pharmacologically. TLR4, downstream of HMGB1, is known to drive PAH development in multiple models (35, 36). Additionally, NF-κB, downstream of TLR4 and MyD88, induces the expression of IL-6, itself sufficient for driving PH development in mice (37). Lastly, an important “gut-lung axis” has been identified, whereby bacterial lipopolysaccharide from the gut stimulate adverse remodeling and inflammation in the lung in PAH and heart failure (38). Together, these systems all converge upon the same signaling players, setting up a tempting possibility whereby breaking this chain via inflammasome inhibition may target multiple important pathogenic pathways in PAH.

Downstream of canonical inflammasome activation, the adaptor protein ASC, and the serine protease, caspase-1, are activated and begin cleaving further downstream substrates. In addition to the data presented above, both of these proteins are involved in PH development in model systems. The double stranded RNA kinase (PKR) is upregulated by Type 1 interferons, and is typically activated during viral infection (39). PKR has been shown to directly bind NLRP3, and loss of PKR inhibits release of IL-1β, IL-18, and HMGB1 (40). Recently, PKR was found to be activated in the pulmonary vessels of both monocrotaline-treated and Sugen-hypoxia treated rats (41). PKR inhibition prevented PH development in these models, and was found to block ASC activation and subsequent release of IL-1β and HMGB1 (41). Mechanistically, PKR was found to promote HMGB1 and cytokine release from endothelial cells, leading to proliferation of cocultured smooth muscle cells (41). These data add a significant regulatory protein to the inflammasome pathway, which can potentially be targeted clinically.

Caspase-1 inhibition has also been explored in PH models. As expected, caspase-1 knockout mice are relatively protected from hypoxic PH, and caspase-1 induced smooth muscle cell proliferation (42). Of note, re-introduction of exogenous IL-1β and IL-18 in caspase-1 knockouts restored the PH phenotype, suggesting that downstream cytokine release is the key role of caspase-1 in this model. Clearly, blockade of canonical inflammasomes can inhibit a variety of important proteins in PH models, and are the subject of substantial clinical and translational inquiry.

While the majority of work in the field focuses on inflammasome activation in macrophage, several other cell types have been shown to also express and activate this pathway, including neutrophils (43), epithelial cells (44), and interestingly for PAH, endothelial cells (45). In acute lung injury models, pulmonary artery endothelial cells were shown to activate inflammasomes and undergo pyroptosis (46). These results establish an intriguing possibility whereby inflammasome activation has an immune effect, as well as a direct effect on the pulmonary vasculature, stimulating cell death, cytokine release, elevated intracellular calcium concentration, and other critical events of known importance in the pathogenesis of PAH. Targeting such a mechanism may, therefore, have pleiotropic effects in a complex disease like PAH.

## Clinical Evidence

Broadly speaking, the importance of inflammation in vascular remodeling in PAH is well-accepted (25, 27, 47). Evidence for the importance of inflammasome activation in PAH patients comes from both biomarkers and interventional studies (Table 1). Indeed, inflammasome activation is broadly applicable to a wide variety of pulmonary diseases (1). In PAH, levels of HMGB1—itself released from pyroptotic cells—are elevated in patients with idiopathic or congenital heart disease-associated PAH (36, 48). Serum IL-1β is elevated in PAH patients and has been shown to correlate with worse outcomes (49). Proof of principle for IL-1β blockade, taken from the preclinical studies detailed above, allowed for the CANTOS trial to examine this approach in patients with atherosclerosis (50). In this large randomized controlled trial, IL-1β blockade with canakinumab decreased both recurrent cardiac events and indices of inflammation. This approach has now been tested in PAH patients, albeit on a smaller scale. Six patients with PAH and right ventricular failure were given anakinra, an IL-1R antagonist. While hemodynamic measures were unchanged over the 3-month study, there was a significant improvement in heart failure symptoms, and a decrease in C-reactive protein levels (51).

Attempts at clinical translation of inflammasome-based therapies will likely grow rapidly in the coming years. Indeed, a variety of drugs are poised for trial, or have themselves entered trials already. The priming step of inflammasome activation largely depends on toll-like receptor-mediated activation of NF-κB (52). To this end, the NF-κB inhibitor bardoxolone, has been in Phase 3 trial in PAH patients, although this trial was recently stopped due to safety concerns surrounding COVID-19 (NCT 02657356). NLRP3 itself can be inhibited by drugs such as MCC-950, which has also shown efficacy in models of cardiac ischemia, aortic disease, and other inflammatory conditions (53, 54). Effective in a variety of models and species, MCC-950 may prove to be an important clinical therapeutic in the future (55). The catalytic action of caspase-1 itself can be targeted by the pro-drug VX-765 [reviewed in (56)]. Further downstream, the effector pore of inflammasome activation, gasdermin D, can be targeted by necrosulfonamide, or the repurposed drug, disulfiram (57, 58). Downstream effectors released from the gasdermin D pore can also be targeted, including the use of anakinra or canakinumab to block IL-1β, or tocilizumab to block IL-6. All of these drugs are undergoing extensive clinical investigation. Together, a wide variety of drugs are under investigation that target inflammasome activity at nearly all steps in the pathway. However, their tolerability, specificity, and overall immunosuppressive side effects will need to be carefully considered and monitored.

## Summary

Inflammasome activation interfaces with PAH across several important elements. Pro-inflammatory cytokines, lytic cell death, leukocyte infiltration, and even endothelial dysfunction can all be stimulated via the activation of inflammasomes in the lung vasculature. Fittingly, this area is an intensive research focus across the continuum of basic translational science and clinical trials. Given the multiple potential points of pharmacological intervention available, inflammasome targeting may prove to be a viable treatment option for PAH patients in the future. Fine-tuning this system, as with all potentially immunosuppressive therapies, will be a key consideration going forward.

Importantly, PAH represents a syndrome arising from a huge range of inciting etiologies. One of the key challenges going forward will be in determining the precise populations that are most likely to benefit from inflammasome-targeted therapy. Important work remains in characterizing biomarkers of inflammasome activation in patients with PAH arising from diverse causes. Several such efforts are underway currently, and have reported novel potential groupings of PAH patients based on sequencing efforts (59, 60). Once a systematic assessment has been made, clinical trials can begin in targeted populations most likely to derive benefit from these translational therapies.

## Author Contributions

AF and NG conceived the concept, wrote the manuscript, and prepared figures. BS conceived the concept, edited the manuscript, and prepared figures. All authors contributed to the article and approved the submitted version.

## Funding

NG was supported by an Early Career Scientist Award from the Canadian Lung Association, AstraZeneca Canada and the CIHR.

## Conflict of Interest

NG received funding from AstraZeneca Canada as part of the CIHR/Canadian Lung Association/AZ Canada Early Clinician Scientist Award. The funder was not involved in the study design, collection, analysis, interpretation of data, the writing of this article or the decision to submit it for publication. The remaining authors declare that the research was conducted in the absence of any commercial or financial relationships that could be construed as a potential conflict of interest.

## Publisher's Note

All claims expressed in this article are solely those of the authors and do not necessarily represent those of their affiliated organizations, or those of the publisher, the editors and the reviewers. Any product that may be evaluated in this article, or claim that may be made by its manufacturer, is not guaranteed or endorsed by the publisher.
